# Uncovering Direct Targets of MiR-19a Involved in Lung Cancer Progression

**DOI:** 10.1371/journal.pone.0137887

**Published:** 2015-09-14

**Authors:** Kumiko Yamamoto, Sachio Ito, Hiroko Hanafusa, Kenji Shimizu, Mamoru Ouchida

**Affiliations:** Department of Molecular Genetics, Graduate School of Medicine, Dentistry and Pharmaceutical Sciences, Okayama University, 2-5-1 Shikata-cho, Kita-ku, Okayama, Japan; National Institutes of Health, UNITED STATES

## Abstract

Micro RNAs (miRNAs) regulate the expression of target genes posttranscriptionally by pairing incompletely with mRNA in a sequence-specific manner. About 30% of human genes are regulated by miRNAs, and a single miRNA is capable of reducing the production of hundreds of proteins by means of incomplete pairing upon miRNA–mRNA binding. Lately, evidence implicating miRNAs in the development of lung cancers has been emerging. In particular, miR-19a, which is highly expressed in malignant lung cancer cells, is considered the key miRNA for tumorigenesis. However, its direct targets remain underreported. In the present study, we focused on six potential miR-19a target genes selected by miRNA target prediction software. To evaluate these genes as direct miR-19a target genes, we performed luciferase, pull-down, and western blot assays. The luciferase activity of plasmids with each miR-19a–binding site was observed to decrease, while increased luciferase activity was observed in the presence of anti-miR-19a locked nucleic acid (LNA). The pull-down assay showed biotinylated miR-19a to bind to AGO2 protein and to four of six potential target mRNAs. Western blot analysis showed that the expression levels of the four genes changed depending on treatment with miR-19a mimic or anti-miR-19a-LNA. Finally, *FOXP1*, *TP53INP1*, *TNFAIP3*, and *TUSC2* were identified as miR-19a targets. To examine the function of these four target genes in lung cancer cells, LK79 (which has high miR-19a expression) and A549 (which has low miR-19a expression) were used. The expression of the four target proteins was higher in A549 than in LK79 cells. The four miR-19a target cDNA expression vectors suppressed cell viability, colony formation, migration, and invasion of A549 and LK79 cells, but LK79 cells transfected with *FOXP1* and *TP53INP1* cDNAs showed no difference compared to the control cells in the invasion assay.

## Introduction

Micro RNAs (miRNAs) are ~22-bp non-coding small RNAs that posttranscriptionally regulate gene expression in a sequence-specific manner [1]. miRNAs are encoded by either their own genes or embedded into introns of the “host” genes and are transcribed by RNA Polymerase II as a part of a long capped and polyadenylated transcript (pri-miRNA) [2]. Pri-miRNAs undergo further processing that involves excision of a hairpin structure along with flanking sequences by a member of RNAse III family Drosha to create pre-miRNA [3–4]. Pre-miRNAs are exported into the cytoplasm by Exportin-5 where they are further cleaved by Dicer that removes terminal loop creating an imperfect RNA duplex [3–5]. One of the strands is preferentially bound by the RNA-induced silencing complex (RISC), which contains Argonaute (AGO) family proteins. Although both strands can become stably associated with AGO family proteins (loading step) only one strand (guide strand; miRNA) is retained by the AGO protein, while the other strand (passenger strand; miRNA*) is degraded. The human AGO proteins (AGO1 to 4) are characterized by a conserved PIWI domain that is structurally similar to the RNAse H. The PIWI domain interacts with the 5’end of mature miRNA and is involved in cleavage of target mRNAs. All four human AGO proteins show remarkably similar structural preferences for small-RNA duplexes: central mismatches (guide position 8–11) promote RISC loading, and mismatches in the seed (guide position 2–7) or 3′-mid regions (guide position 12–15) are required for unwinding [6]. It is difficult for small RNA duplexes bearing mismatches in the seed region to load into AGO proteins [6–12]. On the other hand, the recognition of one miRNA with target mRNAs requires complete or nearly complete matches with the seed region. More than 2,500 miRNAs are reported in humans (GRCh38, http://www.mirbase.org/cgi-bin/browse.pl?org=has), and 30% of human genes are considered to be regulated by miRNAs [13].

Lung cancer is responsible for 19.4% of all cancer-related deaths, which constituted approximately 1.59 million deaths worldwide in 2012 (http://www.who.int/mediacentre/factsheets/fs297/en/). Lung cancer progression is associated with multiple genetic and epigenetic changes that affect gene expression of a wide variety of genes. In particular, alterations in expression of more than two dozen miRNA has been reported in lung cancer patients [14], including recently reported overexpression of the miR-17-92 cluster (oncomiR-1) that encodes, among others, miR-19a and 19b [14]. OncomiR-1 is involved in the regulation of cell survival, proliferation, differentiation, and angiogenesis [15, 16]. Some genes, such as *STAT3* and *MAPK14*, which are involved in tumorigenesis, have been reported as target genes of miR-17-92 in lung cancer cells [17]. MiR-19a, which is highly expressed in malignant lung cancer cells, is considered the key miRNA in tumorigenesis [18]. Cell growth rate and viability differ between lymphomas transfected with wild or mutated miR-19a; therefore, miR-19a might also be associated with cell growth [18]. Moreover, miR-19a activates the Akt-mTOR pathway by repressing the tumor suppressor *PTEN* [18, 19]. Furthermore, *SOCS-1* [20], *THBS1* [21], *IMPDH1*, *NPEPL1* [22], and *TNF-α* are also known as targets of miR-19a [23].

However, as miRNA–mRNA binding depends on seed sequences and imperfect pairing of their strands, miR-19a must have yet-unidentified target genes that influence the onset and progression of lung cancer. In the present study, we identified novel target genes of miR-19a and showed the suppressive ability of the target genes on the growth, migration, and invasion of lung cancer cells.

## Materials and Methods

### Selection of miR-19a target candidate genes

Potential target genes of miR-19a were predicted by using the following miRNA target prediction software: PicTar (http://pictar.bio.nyu.edu), TargetScan (http://targetscan.org), MiRanda (http://cbio.mskcc.org), and miGTS (Kyowa Hakko Kirin Co. Ltd. Tokyo, Japan). The prediction yielded 3,398 genes. To narrow the range of possible miR-19a targets, genes involved in cancer were extracted by search refinement by including more than two words related to cancer (tumor, suppressor, and apoptosis) in the preliminary literature search. Although more than 10 genes remained as miR-19a target candidates, six genes (excluding the genes already reported as miR-19a targets), namely, forkhead box P1 (*FOXP1*), p53-dependent damage-inducible nuclear protein 1 (*TP53INP1*), TNF-α-induced protein 3 (*TNFAIP3*), tumor suppressor candidate 2 (*TUSC2*), SIVA apoptosis-inducing factor 1 (*SIVA1*), and tumor necrosis factor receptor superfamily 12A (*TNFRSF12A*), were selected for this study.

### Cell culture

Human normal lung fibroblast cell line WI-38 and human normal embryonic kidney cell line HEK293 were purchased from American Type Culture Collection (ATCC). Human lung small-cell carcinoma cell lines SBC-5 and LK-79 and human non-small-cell lung cancer cell lines PC3, PC14, LK2, NCI-H23, NCI-H460, NCI-H520, SQ5, Lu65A, Lu-99c, RERF-LCMS, and A549 have been used in our laboratory [24]. HEK293, SBC-5, Lu-65A, PC3, PC14, LK2, NCI-H23, NCI-H460, NCI-H520, SQ5, Lu-99c, and RERF-LCMS were grown in DMEM (Life Technologies, Foster City, CA, USA) or RPMI-1640 (Wako, Osaka, Japan). LK-79 and A549 were grown in mixed DMEM and RPMI-1640 (1:1) supplemented with 10% FBS (Life Technologies), 100 units/mL of penicillin G, and 100 mg/mL of streptomycin (Meiji Seika, Tokyo, Japan). All cells were incubated at 37°C in a humidified atmosphere containing 5% CO_2_.

Exogenous miRNA transfection experiments were performed with miR-19a mimic (1, 5, and 10 nM) (CosmoBio, Tokyo, Japan) using HiPerFect Transfection Reagent (Qiagen, Venlo, Netherlands). The sequences were as follows: miR-19a mimic, sense 5ʹ-p-AUC CGC GCG AUA GUA CGU AUU-3ʹ and antisense 5ʹ-p-UAC GUA CUA UCG CGC GGA UUU-3ʹ; random miRNA (control miRNA), sense 5ʹ-p-UGU GCA AAU CUA UGC AAA ACU GA-3ʹ and antisense 5ʹ-p- UUA GUU UUG CAU AGU UGC AC-3ʹ. The expression of miR-19a was knocked down by transfection with anti-miR-19a locked nucleic acid (LNA) (30 nM; 5ʹ-TCA GTT TTG CAT AGA TTT GCA CA-3ʹ) or a control-LNA oligonucleotide targeting GFP (5ʹ-ATC ACT CTC GGC ATG GAC GAG C-3ʹ) (Gene Design Inc., Osaka, Japan) using HiPerFect Transfection Reagent (Qiagen). The miR-19a target cDNA expression plasmids were transfected into the cells using Lipofectamine 2000 (Life Technologies). The transfected cells were subjected to cell viability assays and RNA extraction 24 h after transfection, and protein extraction 72 h after transfection. Cell viability was determined using a water-soluble tetrazolium salt assay, namely, the 2-[2-methoxy-4-nitrophenyl]-3-[4-nitrophenyl]-5-[2,4-disulfophenyl]-2H-tetrazolium monosodium salt assay (WST-1; Roche Applied Science, Mannheim, Germany).

### Colony formation assay

*FOXP1*, *TP53INP1*, *TNFAIP3*, and *TUSC2* cDNA expression plasmids were transfected into A549 and LK79 cells using Lipofectamine 2000 and selected by G418 (100–400 μg/mL) (Sigma-Aldrich, St. Louis, MO, USA) in 6-well plates. After 3 weeks, the colonies consisting of more than 200 cells were stained with crystal violet. The number of colonies was then counted, and average values were calculated in triplicate wells.

### Cell growth

A549 or LK79 cells transfected with *FOXP1*, *TP53INP1*, *TNFAIP3*, or *TUSC2* cDNA expression plasmid vector were selected by G418 to obtain cells with stable expression. After 3 weeks, the bulk population of G418-resistant cells was collected and used for the cell growth assay, migration assay, and invasion assay. For the cell growth assay, cells with stable expression were cultured at 500 cells/well in a 96-well plate. After 24, 48, and 72 h, the cells were counted using Hoechst 33342 staining and microscopy. Average values of the cells with clearly stained nuclei were calculated in triplicate wells.

### Migration assay

Cells were grown to 95% confluence in a 60-mm plate and scratched with a pipette tip. The floating cells were removed by washing with phosphate-buffered saline (PBS), and microscopic images of the wounds were obtained at 24 h.

### Invasion assay

A Corning Matrigel Invasion Chamber (24-well plate 8.0 micron; Corning, NY, USA) was used for the invasion assay. A549 (1 × 10^5^) or LK79 (2.5 × 10^4^) cells were cultured with serum-free DMEM in the upper chamber separated from the lower chambers, which were supplied with DMEM with 10% FBS by permeable translucent PET membranes. After 24 h, the cells were fixed and stained with Cyto Quick A and B Solution (Muto Pure Chemicals, Tokyo, Japan). Non-invasive cells on the upper side of the membrane were gently removed by wiping, and cells on the lower side were stained and observed by a microscope. The number of invasive cells was counted, and average values were calculated in seven fields per chamber.

### Pull-down assay of target mRNAs of miR-19a

Semi-confluent HEK293 cells on 90-mm culture dishes were washed with cold PBS, harvested by a scraper, and treated with 0.5 mL of 25 mM Tris-HCl (pH 7.5), 70 mM KCl, 2.5 mM EDTA, 0.05% NP-40, 80 U/mL RNase Inhibitor (Life Technologies), and 1× protease inhibitor cocktail (Sigma-Aldrich) on ice for 20 min and centrifuged at 12,000 *×g* for 15 min at 4°C. The supernatant was transferred to a new tube. Biotinylated double-stranded RNA (8 nmoles) of miR-19a (sense 5ʹ-p-UGU GCA AAU CUA UGC AAA ACU GA-biotin-3ʹ and antisense 5ʹ-p-AGU UUU GCA UAG AUU UGC AUA AG-3ʹ) or control random RNA (sense 5ʹ-p-AUC CGC GCG AUA GUA CGU AUU-biotin-3ʹ and antisense 5ʹ-p-UAC GUA CUA UCG CGC GGA UUU-3ʹ) was added to the supernatant (500 μL) and incubated at 4°C for 30 min with 8-rpm shaking and then at 30°C for 1 h with 30-rpm shaking. The extract was incubated at 4°C for 1 h with 10 μL Streptavidin Mutein Matrix (Roche Applied Science), which was pretreated with extraction buffer [250 μg RNase-free BSA and 100 μg yeast tRNA in 500 μL of 25 mM Tris-HCl (pH 7.5), 70 mM KCl, 2.5 mM EDTA, and 0.05% NP-40] for 3 h and washed with the same buffer twice. The Streptavidin/biotin–miRNA/mRNA complex was collected after a spin at 5,000 *×g* for 30 s and washed 5 times at 4°C for 5 min with 8-rpm shaking using 20 mM Tris-HCl (pH 7.4), 400 mM KCl, and 0.5% NP-40. The biotin–miRNA/mRNA complex was eluted with 250 μL of 20 mM Tris-HCl (pH 7.4), 400 mM KCl, 0.5% NP-40, 5 mM biotin, and 80 U/mL RNase inhibitor at 42°C for 5 min. The *PTEN* gene, reported to be an miR-19a target, was used as the positive control, and *DAPK1*, whose sequence did not match with miR-19a seed sequences at the 3ʹ untranslated region (UTR) of its mRNA, was used as the negative control.

### Plasmids

The cDNAs of *FOXP1*, *TP53INP1*, *TNFAIP3*, and *TUSC2* were amplified using PCR with gene-specific primers and human normal cDNA reverse-transcribed from HEK293 mRNA using ReverTra Ace-α kit (Toyobo, Osaka, Japan). The cDNAs were cloned into pBluescript and confirmed by DNA sequencing using the ABI 3100 sequencer (Applied Biosystems, Foster City, CA, USA). FLAG tag sequences were fused at the 5ʹ-end of the cDNA in frame, and they were inserted into the pIRES2-EGFP vector (Clontech, Mountain View, CA, USA) with *Nhe*I and *Sal*I restriction sites. These sequences were reconfirmed by DNA sequencing.

### Luciferase reporter assay

The 3ʹ-UTR fragments containing a possible miR-19a–binding region in the candidate genes were synthesized as oligonucleotides for both strands, which can produce *Xba*I cohesive ends after annealing. The annealed double strands were cloned into the 3ʹ-UTR *Xba*I site of *Renilla* luciferase in the pTK-hRG vector, which was a phRG-B vector (Promega, Madison, WI, USA) bearing luciferase cDNA downstream of the herpes thymidine kinase promoter that we had inserted. The inserted fragments were sequenced, and their orientation and fragment number were determined. For the luciferase assay, the pTK-hRG constructs (180 ng) were co-transfected with the firefly luciferase reporter plasmid pOA-SRα-luciferase (20 ng) as an internal control into HEK293 cells. The pTK-hRG construct with no insert was used as a control. The luciferase activity was measured 48 h after transfection using a dual luciferase reporter assay system (Promega) on a Labosystems Luminoskan RT instrument (Thermo Fisher Scientific, Waltham, MA USA). The relative luciferase activity was calculated by normalizing *Renilla* luminescence to firefly luminescence. For each experimental trial, each sample was assayed in duplicate. The *p*-value was calculated using the two-tailed *t*-test. For the other luciferase assay, the pTK-hRG constructs were co-transfected with anti-miR-19a LNA or control LNA (100 nM) under the same conditions. The pTK-hRG construct bearing a mismatch with the central region of miR-19a seed sequences was used as a negative control.

### Quantitative reverse-transcription PCR

The pulled-down biotin–miRNA/mRNA complex was treated with the guanidium-phenol chloroform extraction procedure with ISOGEN-LS (Nippon Gene, Tokyo, Japan), followed by DNase I (Sigma-Aldrich) and ISOGEN-LS treatment. The mRNA was reverse-transcribed in a total volume of 20 μL using the ReverTra Ace-α kit. The cDNA of the biotin–miRNA/mRNA complex was amplified by PCR in a 20-μL volume containing 0.2 μL of each reverse transcription reaction and 10 μL of the TaqMan 2× Universal PCR Master Mix (Life Technologies) in a 7300 Fast Real-Time PCR System (40 cycles of 95°C for 15 s and 58°C for 5 min). Primers to detect candidate miR-19a target cDNAs were designed flanking a possible miR-19a–binding region in the target mRNA (S1 Table). The TaqMan probe sequences with 5ʹ-FAM and 3ʹ-TAMRA labeling for real-time PCR are shown in S2 Table. The expression level, i.e., cycle threshold (CT) value, of each target mRNA in the pull-down sample with miR-19a–biotin was compared to the CT value of that in the pull-down sample with miR-random–biotin and shown as the relative ratio. Each PCR was performed four times, and the *p*-value was calculated using the two-tailed *t*-test.

For miR-19a expression analysis, total cellular RNA was extracted using ISOGEN (Nippon Gene). The quantitative real-time PCR analysis of miR-19a was performed with a TaqMan MicroRNA Reverse Transcription Kit, TaqMan 2× Universal PCR Master Mix and TaqMan MicroRNA Assay (Life Technologies). Total RNA (10 ng) was reverse-transcribed in a total volume of 15 μL using a TaqMan MicroRNA Reverse Transcription kit. Aliquots of each reverse transcription reaction were amplified by PCR in a 20-μL total volume containing 10 μL of the TaqMan 2× Universal PCR Master Mix. The PCR was performed on a 7300 Fast Real-time PCR System with 50 cycles of 95°C for 15 s and 60°C for 60 s. The expression level (CT value) of miR-19a was normalized to the CT value of a small nuclear RNA, U6B, which was co-amplified as an endogenous control. The ΔCT was calculated as the difference in the CT values between miR-19a and the internal control U6B in one sample. The comparisons of miRNA expression levels were conducted using the ΔΔCT method, where ΔΔCT was the difference in the ΔCT values of a test sample compared to that of the control sample, and 2^-ΔΔCT^ represents the fold change in miRNA expression.

For quantitative real-time PCR analysis of the miR-19a target genes, *FOXP1*, *TP53INP1*, *TNFAIP3*, and *TUSC2* cellular mRNA was reverse transcribed with the ReverTra Ace First Strand cDNA Synthesis Kit (Toyobo), and the cDNA was amplified by PCR in a 20-μL total volume containing 0.2 μL of each reverse transcription reaction and 10 μL of the TaqMan 2× Universal PCR Master Mix (Life Technologies) in a 7300 Fast Real-Time PCR System with 40 cycles of 95°C for 15 s and 58°C for 5 min. The primers and probes used to detect these cDNAs were the same as those for detecting cDNAs of the biotin–miRNA/mRNA complex described above. The CT was calculated as the difference in the CT values between each miR-19a target gene and the β-actin gene in one sample. The expression levels of the target mRNA were also measured using the ΔΔCT method.

### Western blotting analysis

For confirmation of AGO2 protein binding with the biotin–miRNA/mRNA upon *in vitro* pull-down, the complex was combined with gel loading buffer, heated to 95°C for 10 min, and then separated on 12% SDS-polyacrylamide gels and electrotransferred to polyvinylidene difluoride (PVDF) membranes (Life Technologies). Membranes were blocked overnight at 4°C in 3% BSA/PBS and then incubated for 4 h at room temperature with anti-AGO2 monoclonal antibody (No.016-20861, Wako, Osaka, Japan). The filters were washed with PBS/0.05% Tween-20 and then incubated with alkaline phosphatase-conjugated antibodies. The protein signal was visualized using FLA-3000 (Fujifilm, Tokyo, Japan).

For the effect of miR-19a on protein expression, at 72 h after transfection of HEK293 and A549 with the miR-19a mimic or anti-miR-19a-LNA, the protein samples (25 μg) were separated on 8% or 12% SDS-polyacrylamide gels, electrotransferred onto PVDF membranes, and incubated overnight at 4°C with the following antibodies: anti-TP53INP1 (T-17; Santa Cruz Biotechnology, Dallas, TX, USA), anti-FOXP1 (ab16645; Abcam, Cambridge, UK), anti-TNFAIP3 (ab74037; Abcam), anti-TUSC2 (ab70182; Abcam), anti-SIVA1 (ab67620; Abcam), and anti-TNFRSF12A (ITEM-4; Santa Cruz Biotechnology). Anti-β-actin (AC-15; Sigma-Aldrich) was used as a loading control.

### Statistical analysis

The relative ratios in luciferase, RNA expression, cell viability, cell growth, colony formation, and invasion experiments are expressed as the mean values ± SD and were analyzed by the two-tailed *t*-test. A value of *p* < 0.05 was considered statistically significant.

## Results

### Evaluation of miR-19a target candidate genes by the luciferase reporter assay

**Fig 1:**
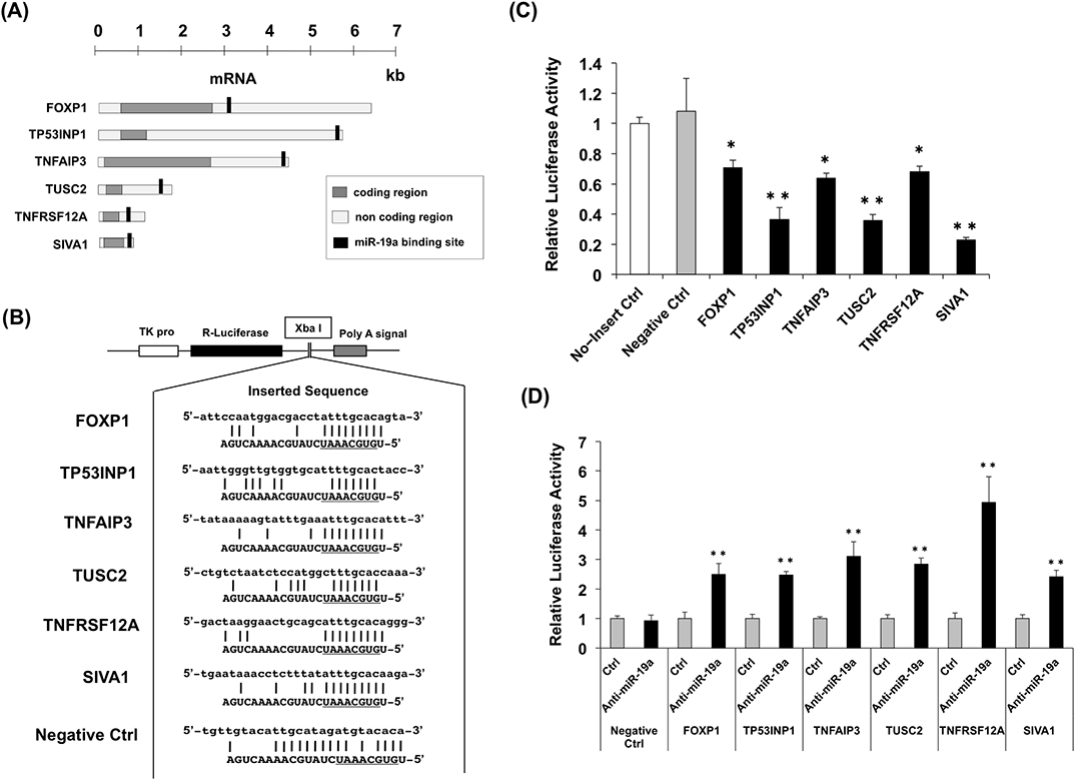
Verification of miR-19a candidate target genes by luciferase assays. (A) Overview of miR-19a target candidate mRNAs. The miR-19a–binding sites identified using PicTar, TargetScan, or MiRanda software are shown in the 3ʹ-untranslated (UTR) region of miR-19a target candidate mRNAs. (B) Construction of luciferase vectors. The miR-19a–binding sites of the candidate genes and negative control sequences (Negative Ctrl) were cloned downstream of the luciferase ORF at the *Xba*I restriction site of the pTK-hRG vector. Sense (*upper*) and antisense (*lower*) strands of complementary sequences indicate the miRNA target site of the mRNA 3ʹ-UTR and miR-19a sequences, respectively. Underlines indicate miR-19a seed sequences. The negative control plasmid has mismatches in the center of miR-19a seed sequences. (C) The luciferase activity of constructs with miR-19a–binding sites was compared with that of the empty vector, which had no insert at the *Xba*I site (No-Insert Ctrl), and was statistically analyzed. (D) Luciferase plasmids with the miR-19a–binding site and negative control plasmid were transfected with anti-miR-19a-LNA or control-LNA (Ctrl). The luciferase activity of cells treated with anti-miR-19a-LNA was compared with that of cells treated with the control-LNA. *, *p* < 0.05 and **, *p* < 0.005 using two-tailed *t*-tests.

We focused on six miR-19a target gene candidates, *FOXP1*, *TP53INP1*, *TNFAIP3*, *TUSC2*, *SIVA1*, and *TNFRSF12A*, which had been selected using miRNA target prediction software. The nucleotide sequences of the possible miRNA-binding site on the 3ʹ-UTR of their mRNAs were obtained from miRNA target prediction software (Fig 1A). To verify whether the miRNA-binding sites are regulated by miR-19a *in vivo*, we constructed luciferase reporter plasmids with each miR-19a binding site in the 3ʹ-UTR of the *Renilla* luciferase gene (Fig 1B). Luciferase reporter plasmids with the sequences mismatched with the miR-19a seed region comprised the negative control. We transfected *Renilla* luciferase reporter plasmids bearing possible miR-19a–binding sites of miR-19a target candidates and internal control firefly luciferase plasmids into HEK293 cells in which miR-19a expression was confirmed. The plasmids were then examined for luciferase activity by the dual-luciferase system. The luciferase activity of all plasmids with the miRNA-binding sites was significantly lower than that of the empty vector control (Fig 1C), suggesting that miR-19a–binding sequences in each miR-19a target candidate mRNA are recognized correctly by endogenous miR-19a in HEK293 cells. Furthermore, we examined the luciferase activity of the plasmids bearing the miRNA-binding sites under miR-19a knockdown by co-transfection with anti-miR-19a LNA. The luciferase activity of all plasmids with the miRNA-binding sites increased significantly in the cells treated with anti-miR-19a LNA as compared with that of the cells treated with control LNA (Fig 1D). These results suggest the possibility that these genes are miR-19a target genes. We then performed other experiments to evaluate the possibility of these being miR-19a target genes.

### Evaluation of candidate genes by an *in vitro* pull-down assay using biotinylated miR-19a

**Fig 2:**
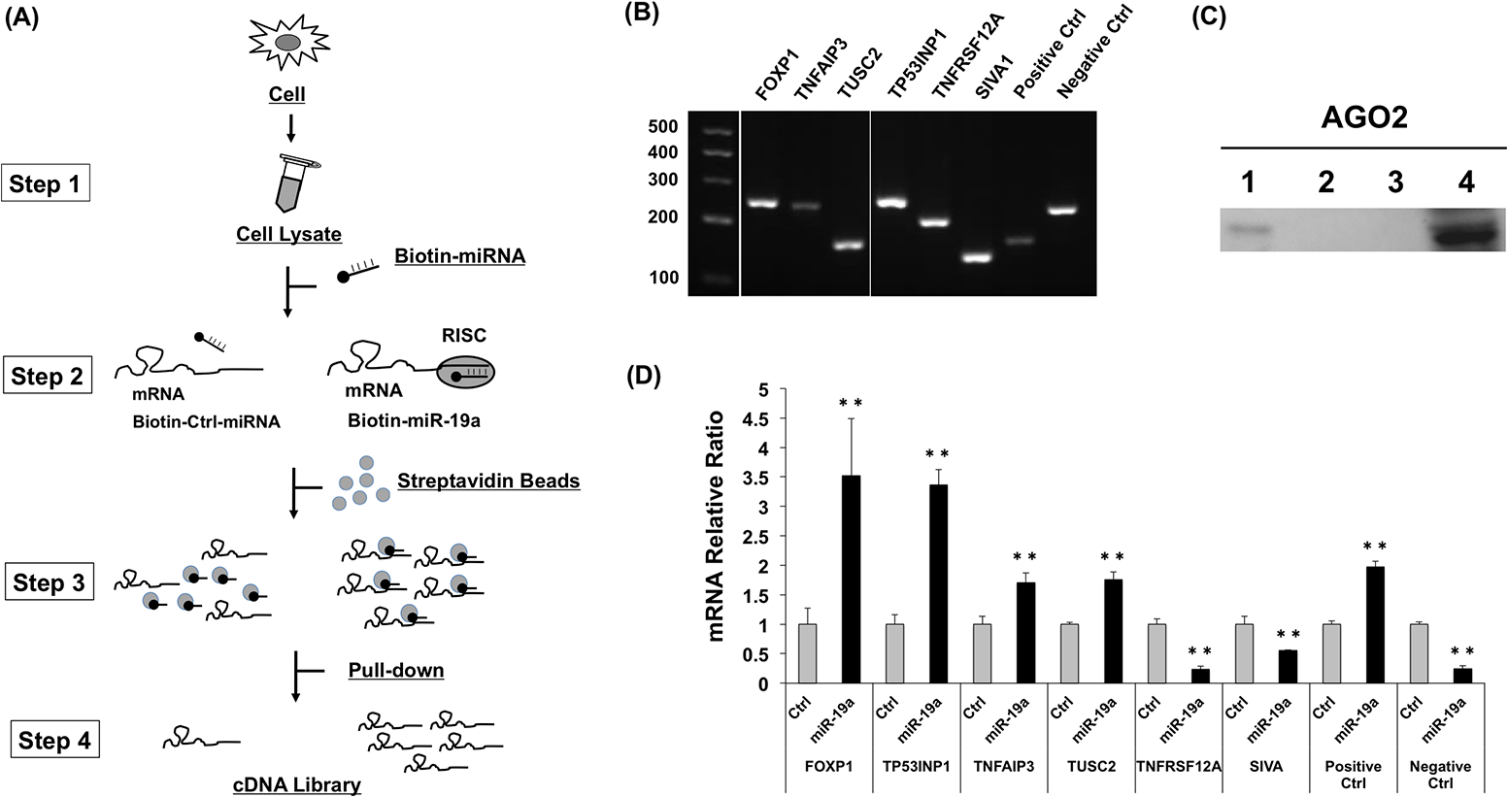
Pull-down assay using biotinylated miRNA. (A) Overview of the *in vitro* pull-down assay. The biotinylated double-stranded miR-19a or control random RNA was incubated in cell extract (step 1) to yield a complex of the biotinylated miRNA single strand with target mRNA and RISC (step 2). The biotinylated miRNA/target mRNA complex was incubated with streptavidin beads and pulled down (step 3). The complex was treated by DNase I and reverse-transcribed (step 4). (B) The expression of miR-19a target candidate mRNAs in HEK293 cells. *PTEN*, known as one of miR-19a target genes, was used as a positive control. The gene, whose sequence did not match with miR-19a seed sequences, was used as the negative control. (C) Confirmation of AGO2 protein in biotinylated miRNA/target mRNA complex by western blotting with the AGO2 antibody. Biotinylated miR-19a (lane 1), biotinylated control random RNA (lane 2), and biotin (lane 3) were used for the pull-down assay and subjected to SDS-polyacrylamide gel electrophoresis and western blotting. Total cell extract was used as the positive control (lane 4). (D) Detection of target mRNAs in biotinylated miRNA/target mRNA complex by real-time RT-PCR. The relative level of each target mRNA in the complex pulled down by using biotinylated miR-19a was compared to that of the complex pulled down by using the biotinylated control random RNA. **, *p* < 0.005 using two-tailed *t*-tests.

MiRNA target genes were recently recovered by a two-step procedure wherein the mRNA/miRNA/FLAG-AGO2 complex was first pulled down with an anti-FLAG antibody, and then, the mRNA/biotinylated miRNA complex was purified by Streptavidin beads in the extract of the cells transfected with biotinylated miRNA and FLAG-AGO2 cDNA expression vector [25]. Therefore, to evaluate our candidate genes as miR-19a targets, we adopted the improved *in vitro* pull-down assay (Fig 2A). At first, the cell extract was mildly prepared from HEK293 cells in which sufficient expression of each candidate mRNA was detected (Fig 2B). The biotinylated double-stranded miR-19a or control random RNA was incubated in cell extract to produce a complex of the biotinylated miRNA single strand with RISC and mRNA. The biotinylated miRNA/mRNA/RISC complex was incubated with Streptavidin beads in the extract, collected by short centrifugation, and eluted with elution buffer containing 5 mM biotin from Streptavidin beads. The biotinylated miRNA/mRNA complex was treated by phenol–chloroform extraction, DNase I, and reverse transcription. Before RT-PCR analysis of the target candidate genes, we confirmed whether the biotinylated miR-19a/mRNA complex contains the RISC component AGO2 by western blotting. The pull-down complex with biotinylated miR-19a exhibited the signal of the AGO2 protein (Fig 2C, lane 1), while no signal was noted for the pull-down complex with biotinylated random RNA (Fig 2C, lane 2), suggesting that biotinylated miR-19a, target mRNA, and AGO2 might form a complex in this pull-down assay. Then, we compared the quantity of candidate target mRNAs in the complexes pulled down using biotinylated miR-19a and control random RNA by quantitative real-time PCR. PCR primers for the candidate target genes were designed to amplify the region (100~300 bp) containing miR-19a–binding sites in their 3ʹ-UTRs (S1 Table). Among the six candidates, *FOXP1*, *TP53INP1*, *TNFAIP3*, and *TUSC2* mRNA levels were significantly higher in the complex pulled down with biotinylated miR-19a than those of the control (Fig 2D). For the confirmation of this pull-down system, we examined a positive control miR-19a target gene, *PTEN*, as well as a negative control gene, whose sequence did not match the miR-19a seed sequences. We detected an increased level of *PTEN* mRNA and decreased level of the negative control gene mRNA in the pull-down complex by using biotinylated miR-19a. Therefore, we focused on evaluating *FOXP1*, *TP53INP1*, *TNFAIP3*, and *TUSC2* genes as miR-19a targets.

### Evaluation of four candidate genes by western blotting

**Fig 3:**
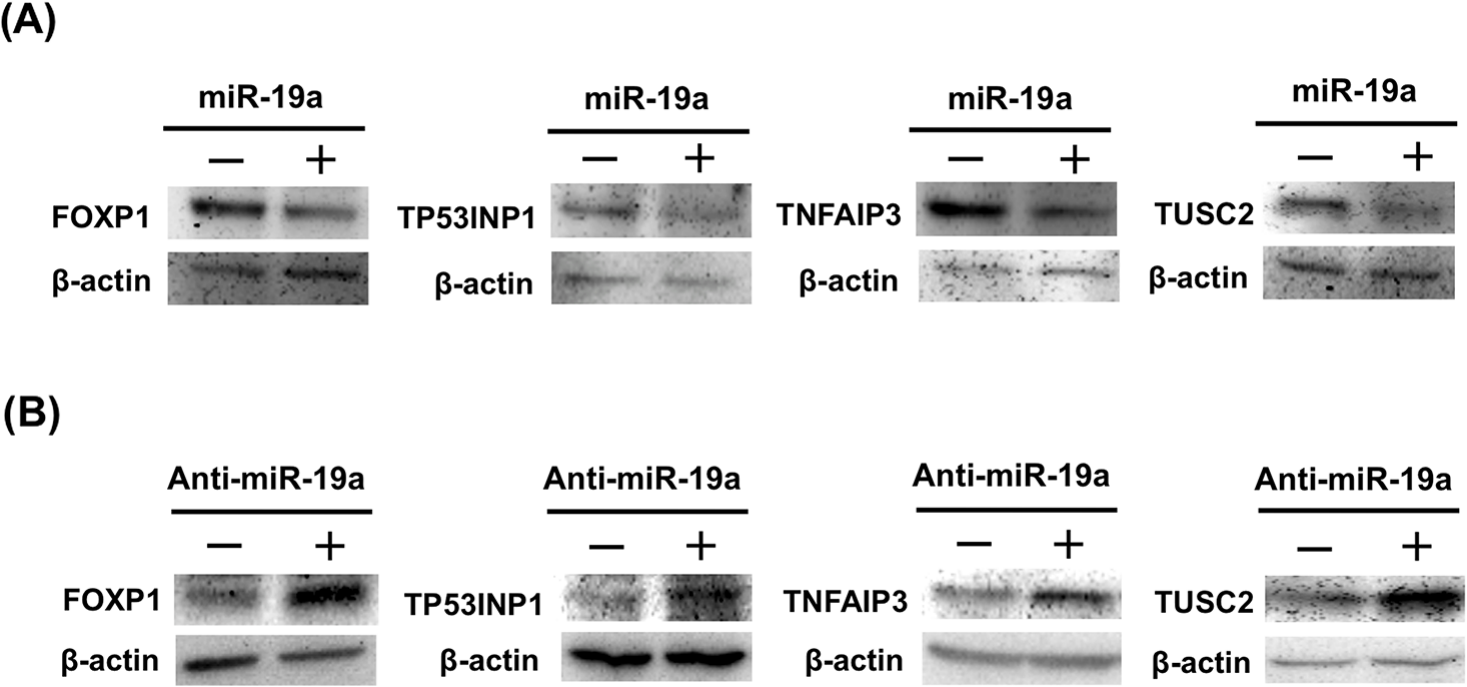
Verification of miR-19a target candidate genes by western blot analysis. (A) Expression analysis of candidate proteins by western blot analysis using proteins of HEK293 cells transfected with miR-19a mimic or control oligo RNA.–, control oligo RNA; +, miR-19a mimic. (B) Expression analysis of candidate proteins by western blot analysis using proteins of HEK293 cells transfected with anti-miR-19a LNA or control LNA.–, control LNA; +, anti-miR-19a LNA.

Genes *FOXP1*, *TP53INP1*, *TNFAIP3*, and *TUSC2* were evaluated as miR-19a targets using another analysis for examining posttranscriptional regulation by miR-19a in the cells. At first, miR-19a mimic or control random miRNA was transfected into HEK293 cells to examine whether miR-19a reduced the expression of the four miR-19a target proteins. By western blotting at 72 h after transfection, decreased expression of the four target proteins was observed in miR-19a mimic–treated cells as compared to that of control miRNA–treated cells (Fig 3A). Secondly, anti-miR-19a LNA or control LNA was transfected into HEK293 cells to knock down endogenous miR-19a, and the expression of the four candidate proteins was analyzed by western blotting. Increased expression of the four target proteins was observed in anti-miR-19a LNA–treated cells as compared to that of control LNA–treated cells (Fig 3B). Taken together, *FOXP1*, *TP53INP1*, *TNFAIP3*, and *TUSC2* were confirmed to be miR-19a target genes. No change in SIVA1 or TNFRSF12A expression was observed in anti-miR-19a LNA–treated cells as compared to that in control LNA–treated cells (S1 Fig).

### Effect of miR-19a in lung cancer cell lines

**Fig 4:**
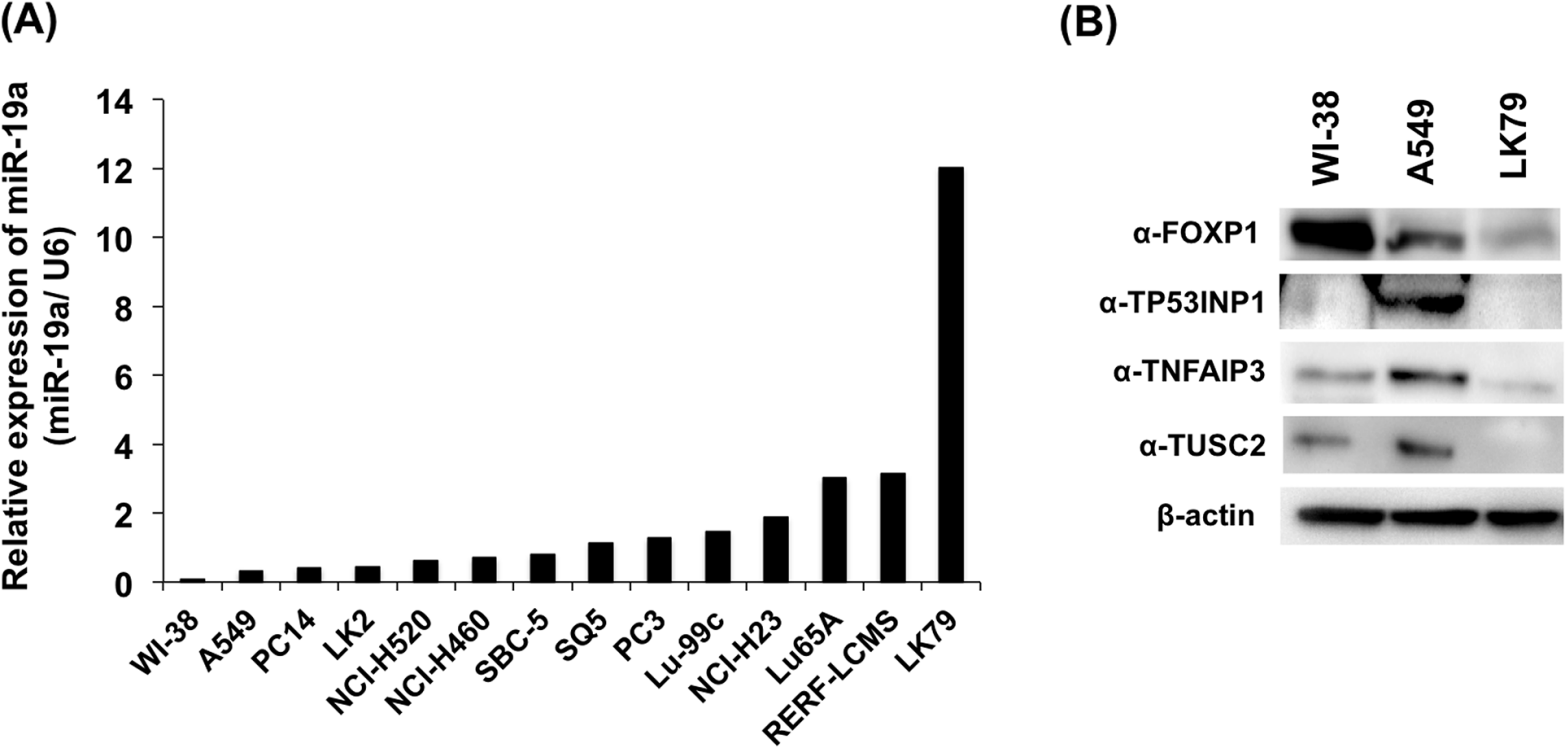
Expression of miR-19a and miR-19a target genes in human lung cancer cell lines. (A) Relative expression of miR-19a in various lung cancer cell lines is shown as compared with that in human normal lung cell line WI-38. (B) Expression of miR-19a target proteins in WI-38, A549, and LK79, as determined by western blot analysis.

To examine the function of the newly identified miR-19a target genes in lung cancer cells, we analyzed the expression level of miR-19a among 13 human lung cancer cell lines using TaqMan RT-PCR analysis. LK79 and A549 showed the highest and lowest level of miR-19a expression, respectively (Fig 4A). LK79 and A549 cells were then selected for the following analyses, to compare the function of these four genes between two lung cancer cell lines showing noticeably different expression levels of miR-19a. Western blotting analysis with antibodies for four miR-19a targets showed that the protein expression level of the four targets was higher in A549 than in LK79 cells (Fig 4B).

**Fig 5:**
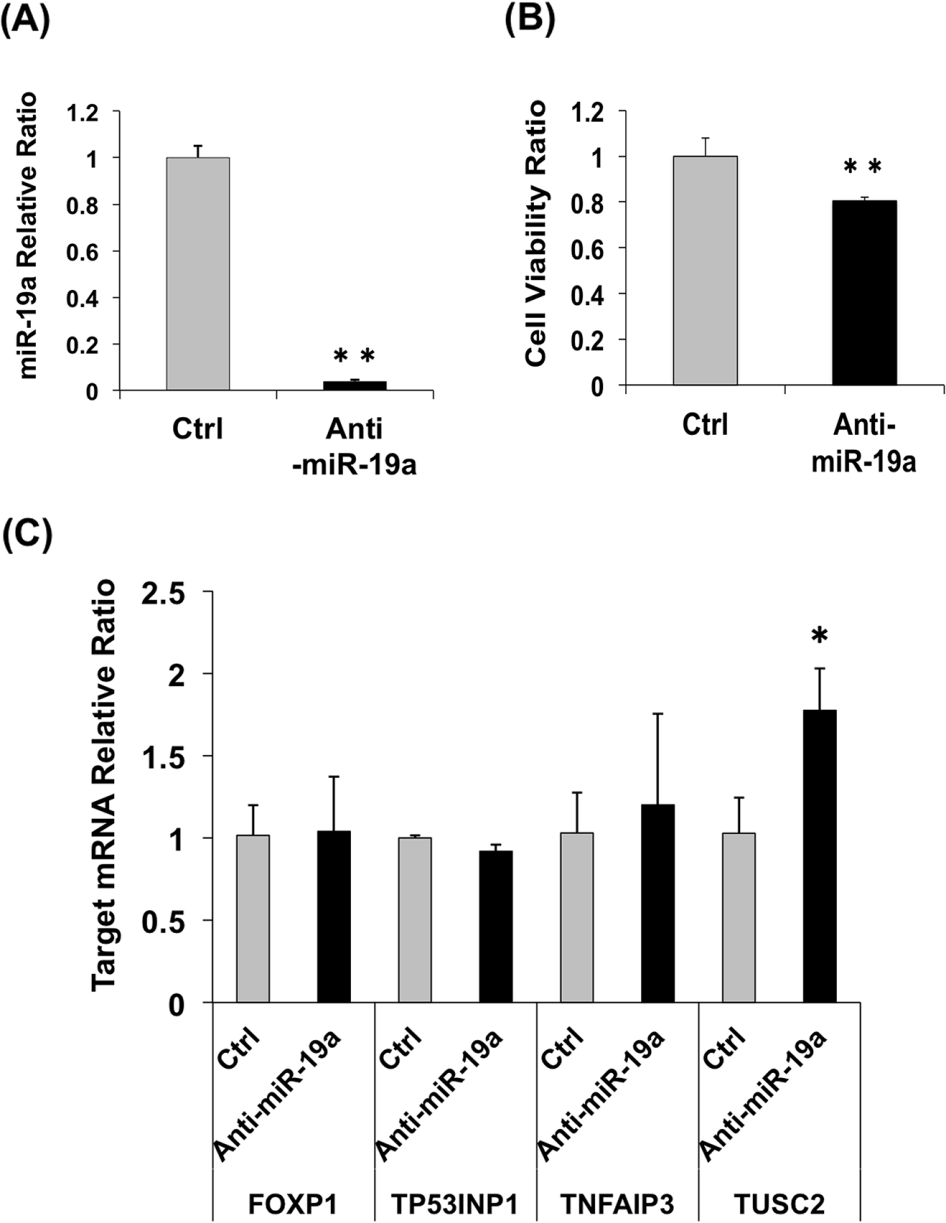
Effect of quantitative change of miR-19a on LK79 cells. (A) Relative expression of miR-19a in LK79 cells 24 h after transfection of anti-miR-19a LNA or control LNA. (B) Cell viability of LK79 cells 24 h after transfection of anti-miR-19a LNA or control LNA. (C) Relative expression of miR-19a target mRNAs in LK79 cells 24 h after transfection of anti-miR-19a LNA and control LNA. *, *p* < 0.05 and **, *p* < 0.005 using two-tailed *t*-tests.

At 24 h after transfection with anti-miR-19a LNA into LK79 cells, the cells treated with anti-miR-19a LNA showed a decrease in the level of miR-19a to one-thirtieth that in the cells treated with control LNA (Fig 5A) and exhibited decreased cell viability (Fig 5B), although the transfection efficiency of LK79 was very low. The mRNA level of *TUSC2* increased significantly, while the mRNA level of *FOXP1*, *TP53INP1*, and *TNFAIP3* showed no significant difference between control and anti-miR-19a LNA–treated LK79 cells (Fig 5C). We could not observe clear differences among the protein levels of the four miR-19a targets by western blotting between control and anti-miR-19a LNA–treated LK79 cells (data not shown).

**Fig 6:**
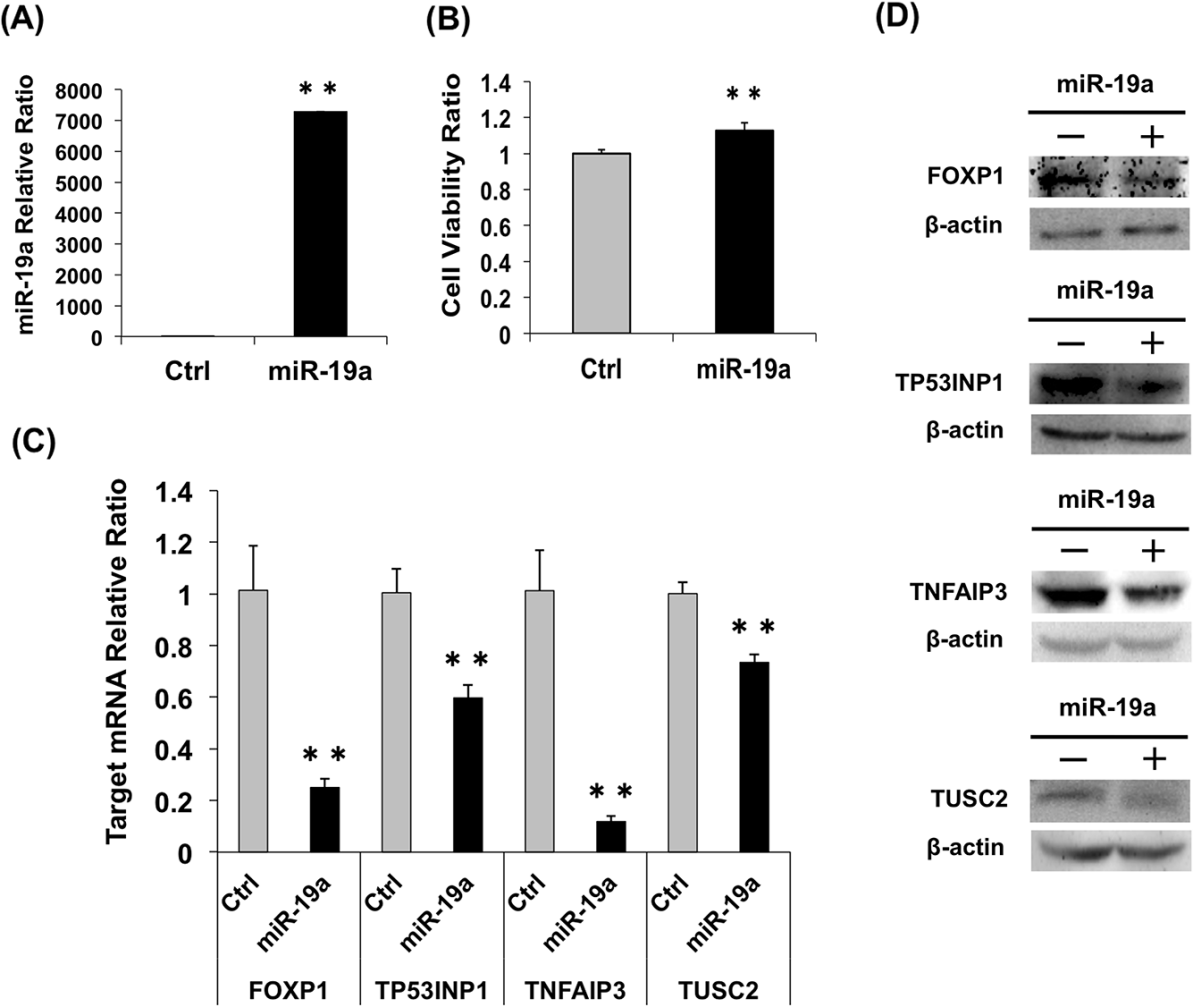
Effect of quantitative change in miR-19a on A549 cells. (A) Relative expression of miR-19a in A549 cells 24 h after transfection of miR-19a mimic or control miRNA (10 nM). (B) Cell viability of A549 cells 24 h after the transfection of miR-19a mimic or control miRNA. (C) Relative expression of miR-19a target mRNAs in A549 cells 24 h after transfection of miR-19a mimic and control miRNA. (D) Relative expression of miR-19a target proteins in A549 cells 72 h after transfection of miR-19a mimic and control miRNA. **, *p* < 0.005 using a two-tailed *t*-test.

At 24 h after transfection of A549 cells, the cells transfected with miR-19a mimic (10 nM) showed an increase of about 7,000-fold of miR-19a as compared with that of the cells treated with control RNA (Fig 6A) and exhibited increased cell viability (Fig 6B). Moreover, the mRNA levels of all four targets decreased significantly in A549 cells treated with the miR-19a mimic compared to control cells (Fig 6C). Western blotting analysis showed a decrease in all four miR-19a target proteins in A549 cells treated with the miR-19a mimic compared to those of the cells treated with control miRNA (Fig 6D). Furthermore, the cells transfected with the miR-19a mimic (1 and 5 nM) showed an increase of about 300- and 1,700-fold, respectively, in miR-19a (S2 Fig). Then, the mRNA levels of all four targets decreased in a dose-dependent manner in A549 cells treated with the miR-19a mimic. The levels of four miR-19a target proteins in A549 cells treated with the miR-19a mimic tended to be lower than those in cells treated with control miRNA.

### miR-19a target genes suppress cell viability, migration, and invasion of lung cancer cells

**Fig 7:**
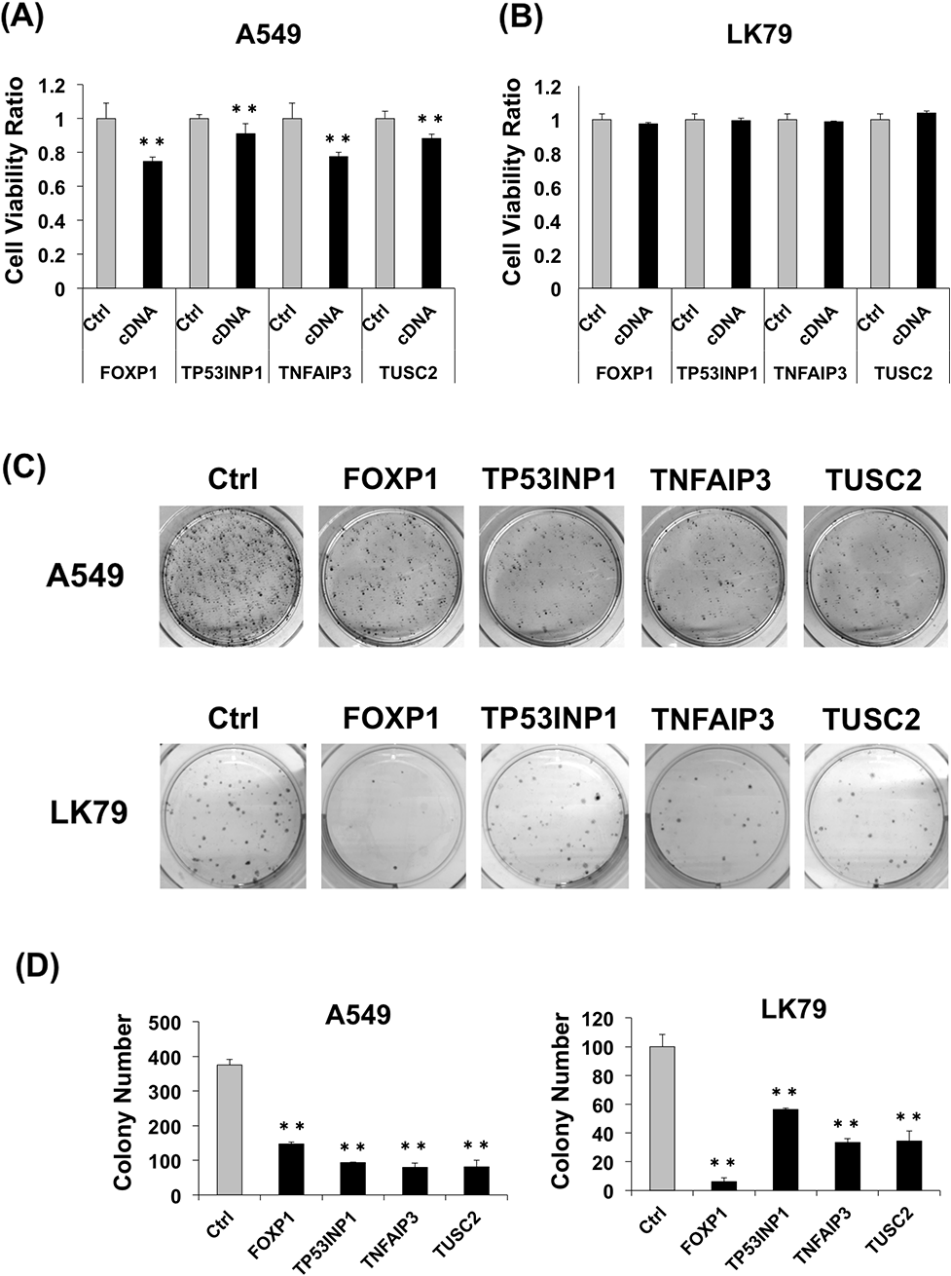
Effect of miR-19a target genes on lung cancer cells. (A) Relative cell viability of A549 cells 24 h after transfection of expression plasmids with miR-19a target gene cDNAs. (B) Relative cell viability of LK79 cells 24 h after transfection of expression plasmids with miR-19a target gene cDNAs. (C) Colony formation of A549 cells (*upper*) and LK79 cells *(lower)* 3 weeks after transfection with miR-19a target cDNA plasmids and G418 selection. Each assay was performed in 3 wells of 6-well plates, and the representative images of the results are shown. (D) Average colony number of A549 cells (*left*) and LK79 cells *(right)* in the colony formation assay. **, *p* < 0.005 using a two-tailed *t*-test.

Expression plasmids for the miR-19a target proteins FOXP1, TP53INP1, TNFAIP3, and TUSC2 fused with the FLAG tag were constructed, and the protein expression derived from the plasmids was confirmed by western blotting using the anti-FLAG antibody (S3 Fig). The expression plasmids were transfected into A549 or LK79 cells, and cell viability was measured 24 h after the transfection. A549 showed a significantly decreased viability in all dishes with the miR-19a target cDNA plasmids compared to the control (Fig 7A), while there was no significant difference in the cell viability of LK79 (Fig 7B). Therefore, to exclude the effect of the low transfection efficiency of LK79, colony-formation assay was performed to examine the effect of the target genes on tumor cell growth. The number of colonies after selection using G418 was smaller in A549 and LK79 cells transfected with the cDNA plasmids as compared to those of the control (Fig 7C and 7D), although the transfection efficiency of LK79 was poorer than that of A549.

**Fig 8:**
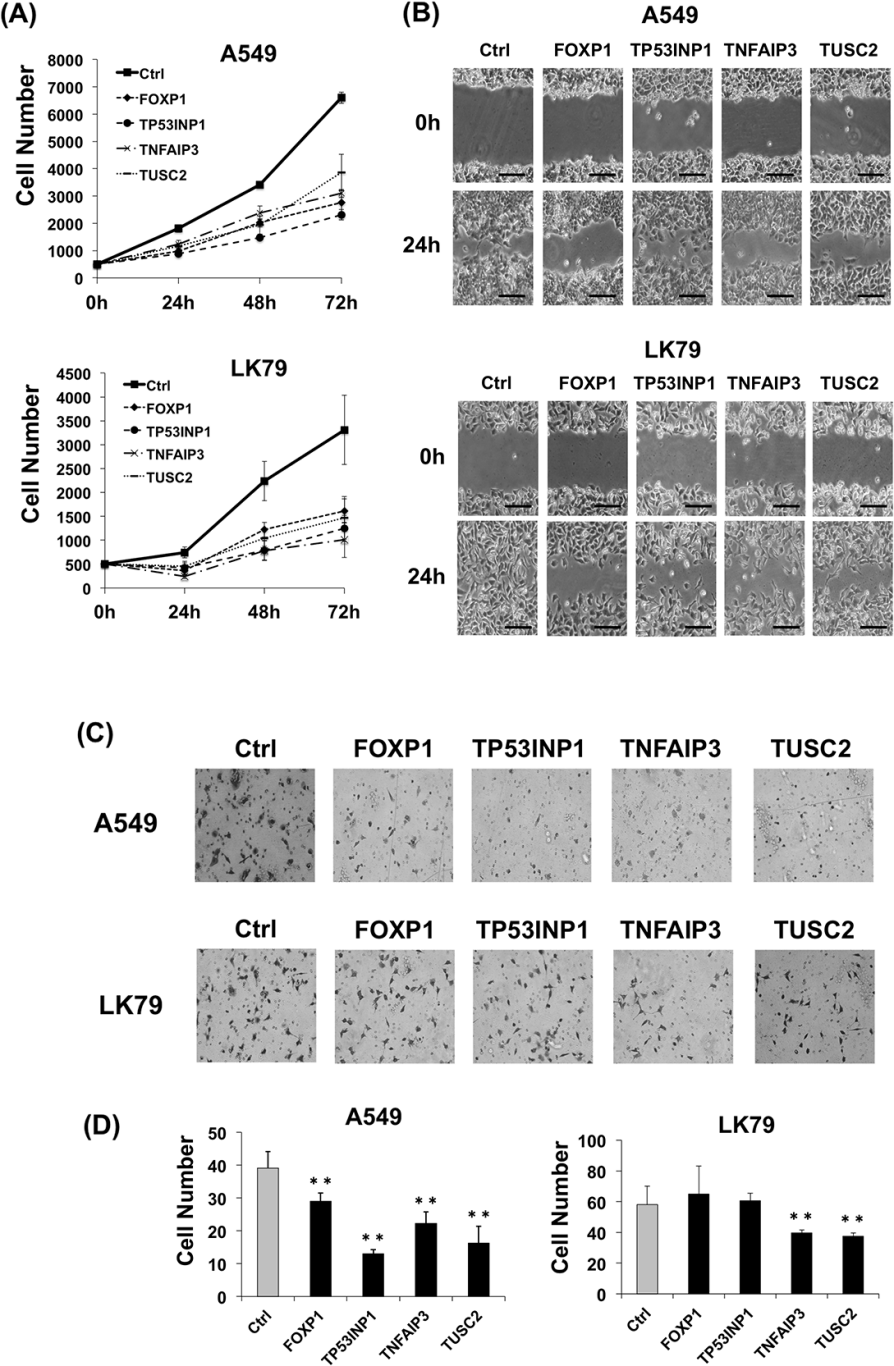
Stable cell lines with miR-19a target genes exhibit suppression of cell growth, migration, and invasion. (A) Cell growth of G418-resistant stable cells of A549 *(upper)* and LK79 *(lower)* transfected with miR-19a target cDNA expression vectors or empty vector (Ctrl). Cells were counted using Hoechst 33342 staining at 24, 48, and 72 h. The number of each miR-19a target expression cell was significantly less than control cells at 24, 48, and 72 h. (B) Migration assays used A549 *(upper)* and LK79 *(lower)* stable cells with miR-19a target genes. Scale bar, 100 μm. (C) *In vitro* invasion assay used A549 *(upper)* and LK79 *(lower)* stable cells with miR-19a target genes. (D) Average number of invading A549 *(left)* and LK79 *(right)* stable cells with miR-19a target genes for the invasion assay shown in (C). **, *p* < 0.005 using a two-tailed *t*-test.

After A549 or LK79 cells were transfected with the expression plasmids and selected with G418, G418-resistant cells were collected and used for examining the effect of the target genes on cell growth, migration, and invasion abilities. Cell count assays at 24, 48, and 72 h showed significantly decreased cell growth in all the cDNA-transfected A549 and LK79 cells as compared to the control cells with the empty plasmids (Fig 8A). All A549 and LK79 cells transfected with the cDNA showed decreased migration as compared with the control cells by an *in vitro* migration assay. The representative images of the results are shown in Fig 8B. In an *in vitro* invasion assay, the cDNA-transfected A549 cells showed significantly decreased invasion in all four targets as compared with the control cells (Fig 8C and 8D). The LK79 cells transfected with *TNFAIP3* and *TUSC2* genes showed significantly decreased invasion, while the LK79 cells transfected with *FOXP1* and *TP53INP1* showed no difference compared to the control cells (Fig 8C and 8D).

## Discussion

Recently, in addition to some reports that miR-19a represses the expression of cell signaling pathway genes such as *PTEN* or *TNF-α* [18, 19, 23], the overexpression of miR-19a in gastric cancer has been demonstrated to promote the epithelial–mesenchymal transition by activating the PI3K/AKT pathway [26]. Furthermore, patients with multiple myeloma and low miR-19a levels have been found to have an improved response to bortezomib compared to those with high miR-19a expression, and they experience a significantly extended survival upon bortezomib-based therapy [27]. Although there are many reports on the role of miR-19a and malignant tumors, miR-19a has been considered a clinical biochemical marker for acute myocardial infarction (AMI) diagnosis because circulating miR-19a expression is high in AMI patients [28]. Thus, miR-19a also has potential applications in basic cancer studies, therapeutic strategies and decision-making, and as a biochemical marker for prognosis prediction of many kinds of malignant tumors.

In the present study, we investigated the effect of miR-19a on lung cancer cells by transfecting an miR-19a mimic or anti-miR-19a LNA into cells with high (LK79 cells) or low (A549 cells) miR-19a expression. The changes in the viability of the transfected cells demonstrated that miR-19a participates in the regulation of the cell cycle as dictated by miR-19a levels, and these cells are responsible for the difference in miR-19a quantities (Figs 5B and 6B). However, the transfection efficiency of LK79 was very low compared to that of A549. The miR-19a relative ratio showed a 1/30-fold decrease in LK79 transfected with anti-miR-19a LNA, while the miR-19a relative ratio showed a drastic 7,000-fold increase in A549 transfected with the miR-19a mimic. No significant difference in cell viability was observed in LK79 transfected with the target cDNA expression vectors (Fig 7B), suggesting that low transfection efficiency of LK79 might be one of the causes. Fig 4 shows differences in the amounts of miR-19a and the four target proteins between LK79 and A549 cells. Knockdown of miR-19a to 1/30 of the control level by LNA in LK79 cells (Fig 5A) may be expected to lead to increase in mRNA and protein expression of the target genes. However, the expression ratio of miR-19a and the target mRNAs might be extremely different in the two cell lines. In the different cell lines, which have differing expression ratios of miRNA and the target mRNAs, the effective knockdown ratio of miRNA to target mRNAs may be different and may vary according to the cell line. In LK79 cells, miR-19a might be expressed at much more higher levels than the target mRNAs. Therefore, the knockdown of miR-19a to 1/30 of the control level might not have much effect on the increase in the target mRNAs in LK79 cells, as seen in most cases in Fig 5C. However, these data do not preclude these four genes as miR-19a targets because significant differences were observed in target mRNA and protein analyses of A549 transfected with the miR-19a mimic (Fig 6C and 6D) and in cell viability of A549 transfected with the target cDNA expression vectors (Fig 7A, S4 Fig).

To evaluate the effect of *FOXP1*, *TP53INP1*, *TNFAIP3*, and *TUSC2* on LK79 cells independently from transfection efficiency, G418-resistant LK79 and A549 colonies were collected and used for colony formation, cell growth, cell migration, and cell invasion assays. All four miR-19a target genes showed a suppression effect for colony formation, cell growth, and migration in both LK79 and A549 lines **(** Fig 7C and 7D; Fig 8A and 8B). In the invasion assay, *TNFAIP3* and *TUSC2* showed a suppression effect in LK79, while the 4 target genes showed suppression of invasion in A549 **(** Fig 8C and 8D **)**. The underlying reason might be the difference in malignancy between A549 (non-small cell lung carcinoma, NSCLC) and LK79 (SCLC, small cell lung carcinoma). It is also reported that LK79 cells overexpress chemokine CCL2, which promotes migration and metastasis [29, 30]. Another reason might be that strong overexpression of miR-19a in LK79 caused decreased protein level of not the targets but also other unidentified targets of miR-19a. Therefore, decreased expression of some other target genes might affect the suppressive effect of invasion in LK79.

We used an *in vitro* pull-down assay for screening the target genes with biotinylated miR-19a and HEK293 cells. HEK293 transfected with the miR-19a mimic primarily showed increased cell viability, and HEK293 transfected with anti-miR-19a LNA showed decreased cell viability (data not shown), suggesting that HEK293 was available for evaluation assays of miR-19a target genes. In the assay, when *DAPK1*, whose sequence did not match with that of the seed region of miR-19a, was used as a negative control, the relative ratio of *DAPK1* mRNA was significantly lower than that of the positive control *PTEN* in mRNA samples pulled down by using biotinylated miR-19a (Fig 2D). These results suggest that this assay is suitable for searching miRNA target genes.

The functions of FOXP1, TP53INP1, TNFAIP3, and TUSC2 and the scope or availability of gene-based therapies for certain diseases are described in the following paragraphs.

*TUSC2* maps to the human chromosome 3p21.3 region, which is frequently lost or reduced in lung cancer [31]. *TUSC2* is reported to decrease cell growth, migration, invasion, and colony formation and to be able to lead tumors to apoptosis [32]. A loss or reduction in TUSC2 protein expression is associated with poor overall survival. It has been shown to function as a potent proapoptotic factor. The first human systemic gene therapy clinical trial of *TUSC2* has also been reported recently [33–36]. Although multiple miRNAs downregulate TUSC2 protein through the mRNA 3ʹ-UTR, *pseudogene TUSC2P* mRNA has the same 3ʹ-UTR as TUSC2 mRNA. Consequently, *pseudogene TUSC2P* promotes *TUSC2* function by binding with multiple miRNAs [32]. MiR-19a would be one of the multiple miRNAs regulating TUSC2 through the pseudogene, because we found possible miR-19a–binding sequences on the 3ʹ-UTR of *pseudogene TUSC2P*.

FOXP1 is a forkhead transcriptional repressor that regulates the development of lung, lymphocytes, monocyte differentiation, and macrophage activities. In cancers, FOXP1 can act as a tumor suppressor [37, 38]. Genetic aberrations of Foxp1 loci at chromosome 3p14.1 have been reported in many cancers including lung cancer and neuroblastoma. Indeed, deletions at 3p have been observed in the majority of small (SCLCs) and of non-small (NSCLCs) cell lung cancers and the extent of deletions correlated with tumor progression [39]. Similarly, the loss of heterozygosity at 3p has been reported in neurobalstomas where it correlated with poor prognosis. Taking together these observations led to the hypothesis that *FOXP1* is a tumor suppressor gene. Indeed re-introduction of *FOXP1* into neuroblastoma cells inhibited cell proliferation and colony formation in soft agar, and resulted in cell cycle arrest and apoptosis [38, 40].

*TP53INP1* is a p53-inducible tumor suppressor gene. The protein complex interacting with TP53INP1 is shown to phosphorylate Ser46 of p53, leading cells to apoptosis by double-stranded breaks in DNA [41–42]. *TP53INP1* is also targeted by miR-155, a likely oncomiR, in esophageal squamous cell carcinoma [43] and liver cancer stem cells [44]. Our results suggest that miR-19a participates in the progression of lung cancer through *TP53INP1* downregulation.

*TNFAIP3* was identified as a gene whose expression is rapidly induced by TNF-α. This gene encodes a zinc finger protein that has both ubiquitin ligase and deubiquitinase activities. TNFAIP3 inhibits the activity of NF-κB, which strongly facilitates the tumor progression through MMP9 expression, and represses cell division and inflammation [45–49]. As *TNF-α* is a target of miR-19a [23], and our results suggest that *TNFAIP3* is a target of miR-19a, miR-19a might affect TNF-α signaling.

Our results revealed that miR-19a downregulates the target genes *FOXP1*, *TP53INP1*, *TNFAIP3*, and *TUSC2* and that these genes might play important roles in the tumorigenesis of lung cancer; however, the invasion inhibition potency was found to differ among the four genes. Our study had a limitation in that it involved *in vitro* analyses for identifying miRNA target genes based on cultured cells. Therefore, further studies will be required for determining the association of the target genes with tumor malignancy and/or miR-19a expression in clinical studies using pathological specimens and tissues from patients with lung cancer. Information regarding the mechanisms underlying tumorigenesis mediated by the target genes may help provide promising potential targets for effective diagnostic applications and targeted therapies for lung cancer.

## Supporting Information

S1 Fig Analysis of SIVA1 and TNFRSF12A proteins by western blot analysis. Expression of SIVA1 and TNFRSF12A was analyzed by western blotting using proteins from HEK293 cells transfected with anti-miR-19a LNA or control LNA.–, control LNA; +, anti-miR-19a LNA.
(TIF)

S2 Fig Effect of quantitative change in miR-19a on A549 cells. (A) Relative expression of miR-19a in A549 cells at 24 h after transfection of miR-19a mimic or control miRNA (1 and 5 nM). (B) Relative expression of miR-19a target mRNAs in A549 cells at 24 h after transfection of miR-19a mimic and control miRNA. (C) Relative expression of miR-19a target proteins in A549 cells at 48 h after transfection of miR-19a mimic and control miRNA. *, *p* < 0.05; **, *p* < 0.005 using a two-tailed *t*-test.
(TIF)

S3 Fig Protein expression of cDNA expression plasmids coding miR-19a target proteins fused with FLAG tag peptides. At 72 h after transfection of HEK293 with cDNA expression plasmids (*right*) or empty plasmid (*left*), the protein samples (25 μg) were analyzed by western blotting using the anti-FLAG antibody (*upper*) and anti-β-actin antibody (*lower*).
(TIF)

S4 Fig Cell growth of a single colony of G418-resistant A549 cells with each miR-19a target gene. *FOXP1*, *TP53INP1*, *TNFAIP3*, and *TUSC2* cDNA expression plasmids and empty plasmids were transfected into A549 cells and selected with G418. Single colonies were isolated 3 weeks after transfection and used for the cell growth assay. After 24, 48, and 72 h, the cells were counted using Hoechst 33342 staining and microscopy. Average values of the cells with clearly stained nuclei were calculated in triplicate wells.
(TIF)

S1 Table Primers Used to Detect miR-19a Target cDNA. (TIF)

S2 Table TaqMan Probe Sequences for Real-Time PCR. (TIF)
