# Supplementary figures and images for: Uncovering Direct Targets of MiR-19a Involved in Lung Cancer Progression
Source: PLoS One. 2015 Sep 14;10(9):e0137887. doi: 10.1371/journal.pone.0137887 (PMC4569347; doi:10.1371/journal.pone.0137887)

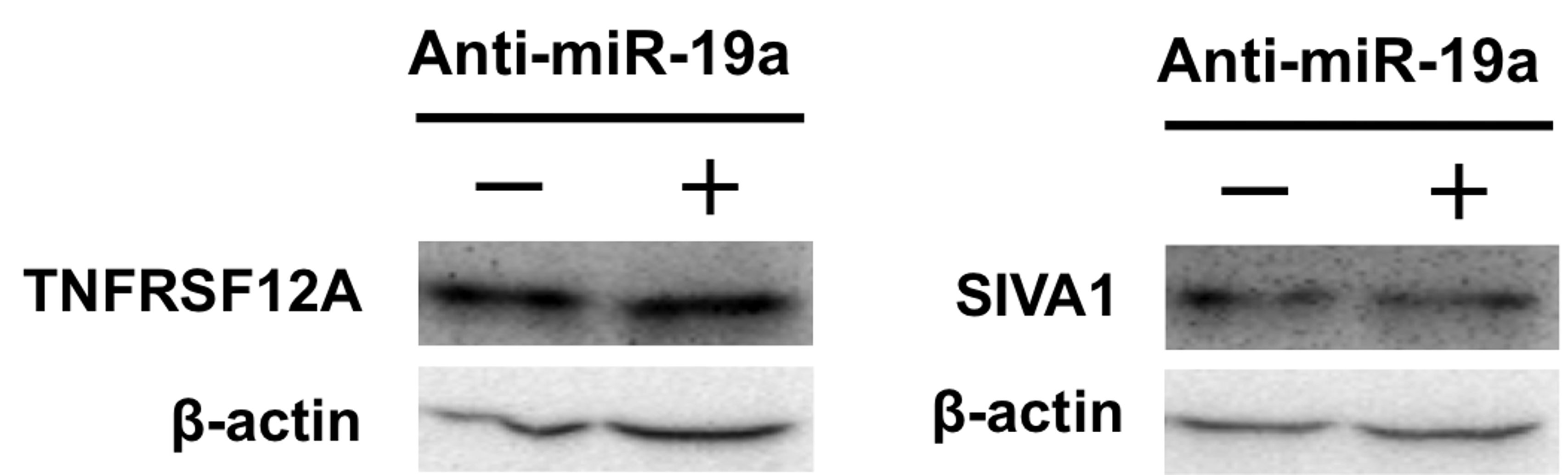

Supplement: S1 Fig — Expression of SIVA1 and TNFRSF12A was analyzed by western blotting using proteins from HEK293 cells transfected with anti-miR-19a LNA or control LNA.–, control LNA; +, anti-miR-19a LNA. (TIF) [file pone.0137887.s001.tif]

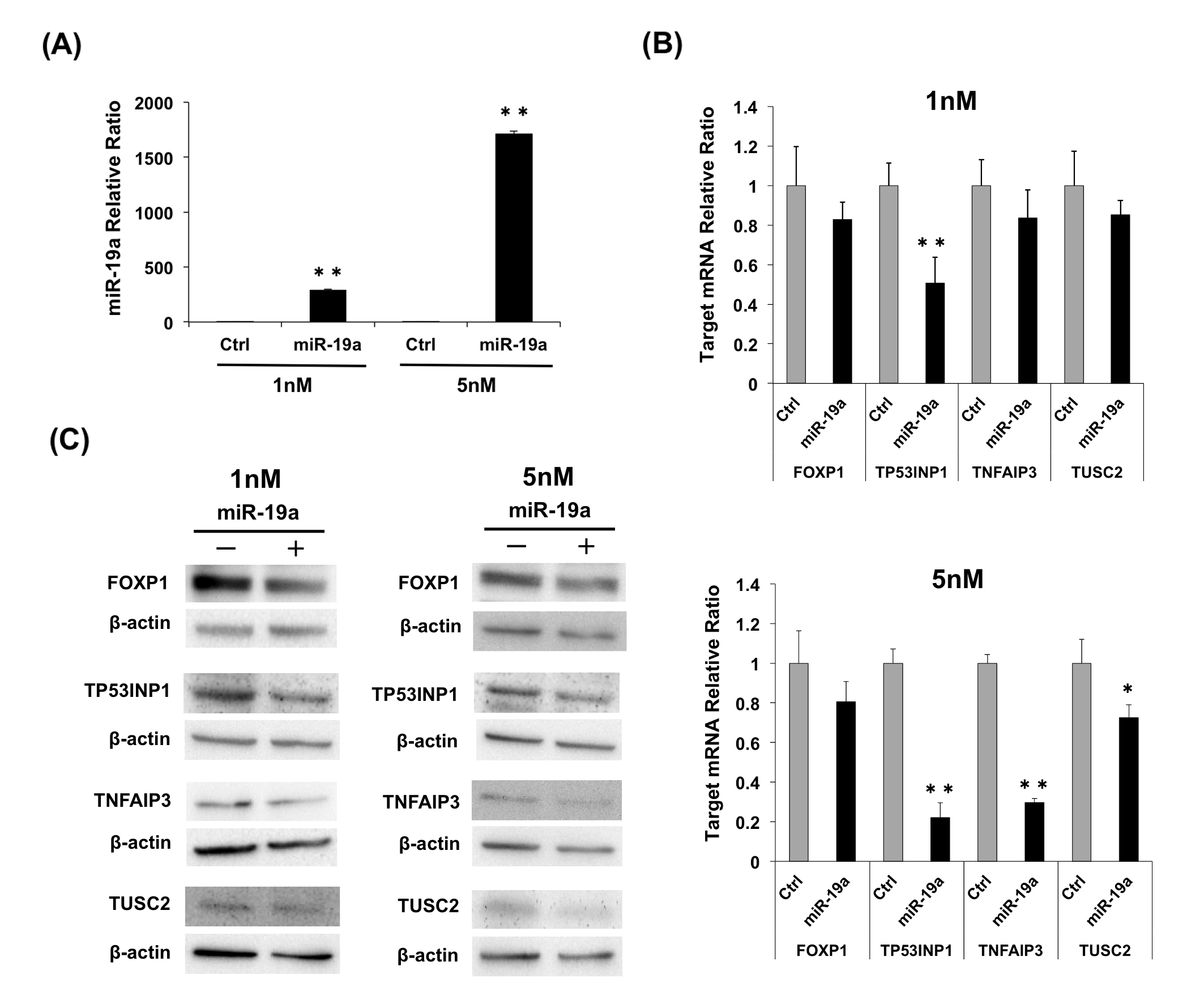

Supplement: S2 Fig — (A) Relative expression of miR-19a in A549 cells at 24 h after transfection of miR-19a mimic or control miRNA (1 and 5 nM). (B) Relative expression of miR-19a target mRNAs in A549 cells at 24 h after transfection of miR-19a mimic and control miRNA. (C) Relative expression of miR-19a target proteins in A549 cells at 48 h after transfection of miR-19a mimic and control miRNA. *, p < 0.05; **, p < 0.005 using a two-tailed t-test. (TIF) [file pone.0137887.s002.tif]

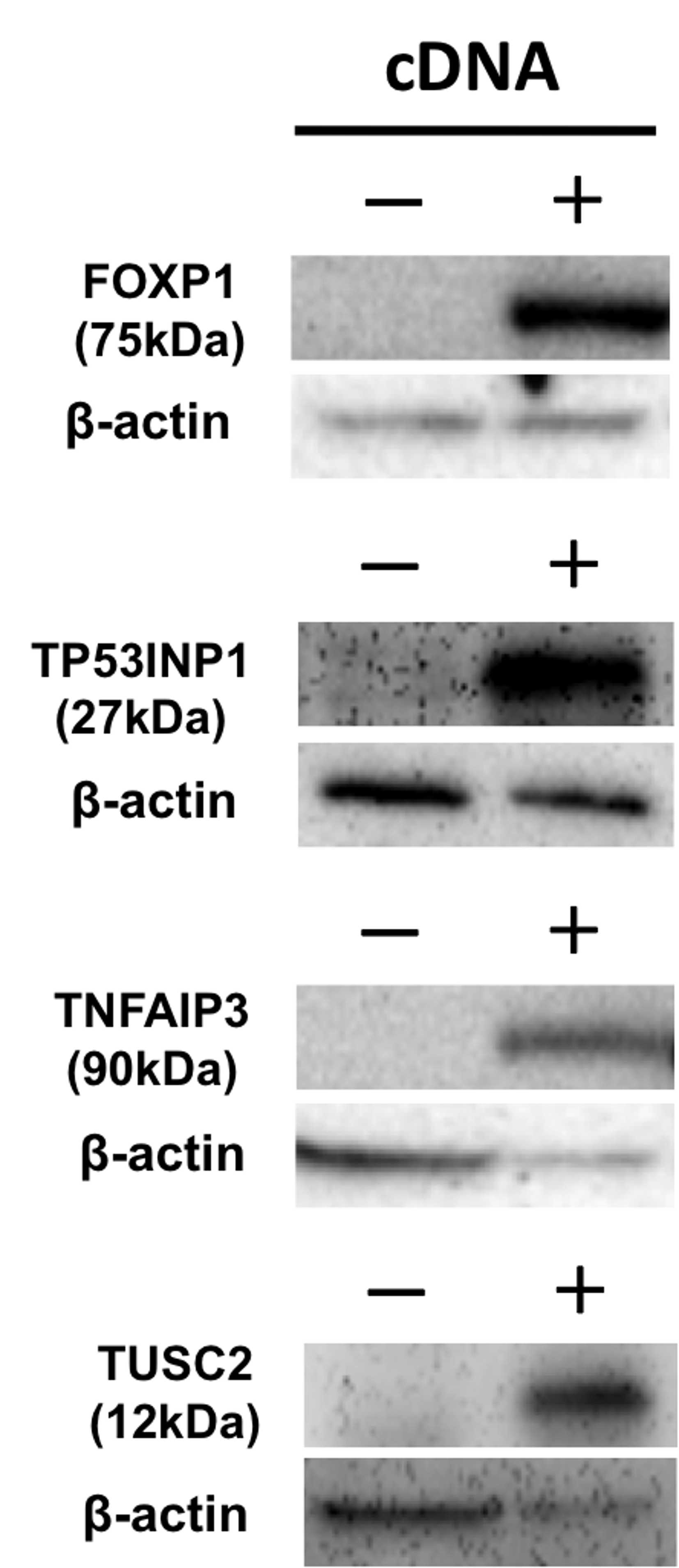

Supplement: S3 Fig — At 72 h after transfection of HEK293 with cDNA expression plasmids (right) or empty plasmid (left), the protein samples (25 μg) were analyzed by western blotting using the anti-FLAG antibody (upper) and anti-β-actin antibody (lower). (TIF) [file pone.0137887.s003.tif]

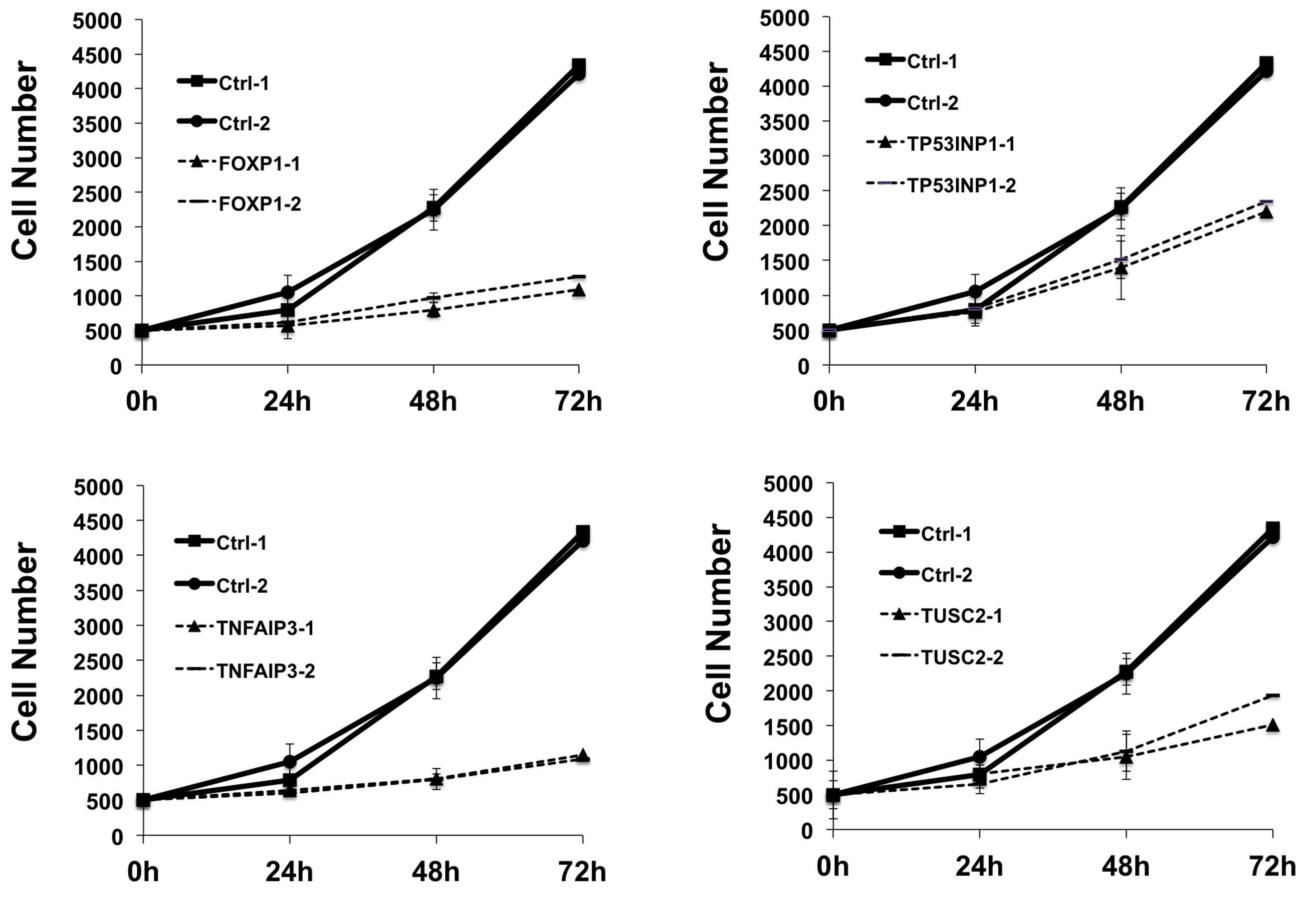

Supplement: S4 Fig — FOXP1, TP53INP1, TNFAIP3, and TUSC2 cDNA expression plasmids and empty plasmids were transfected into A549 cells and selected with G418. Single colonies were isolated 3 weeks after transfection and used for the cell growth assay. After 24, 48, and 72 h, the cells were counted using Hoechst 33342 staining and microscopy. Average values of the cells with clearly stained nuclei were calculated in triplicate wells. (TIF) [file pone.0137887.s004.tif]

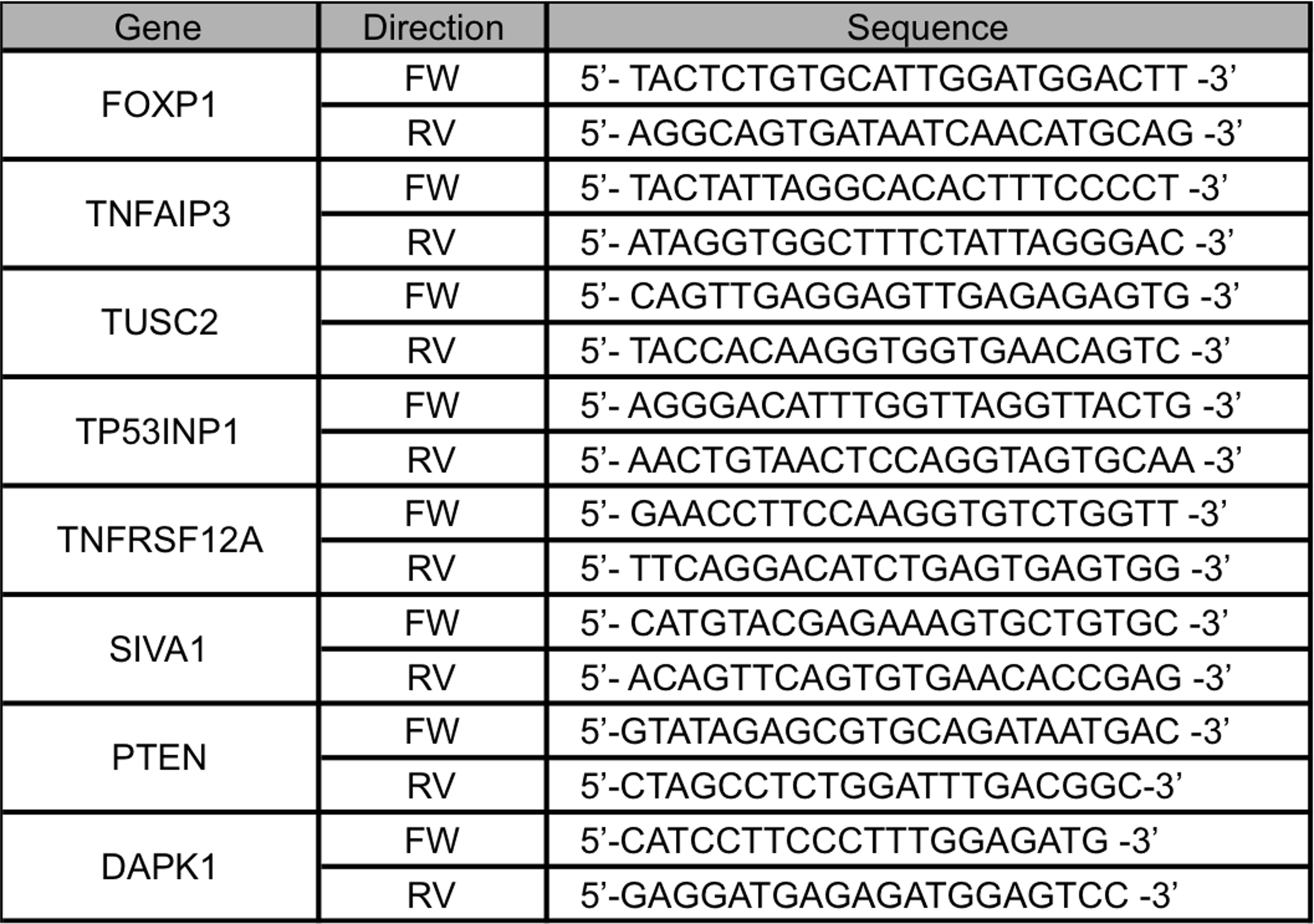

Supplement: S1 Table — (TIF) [file pone.0137887.s005.tif]

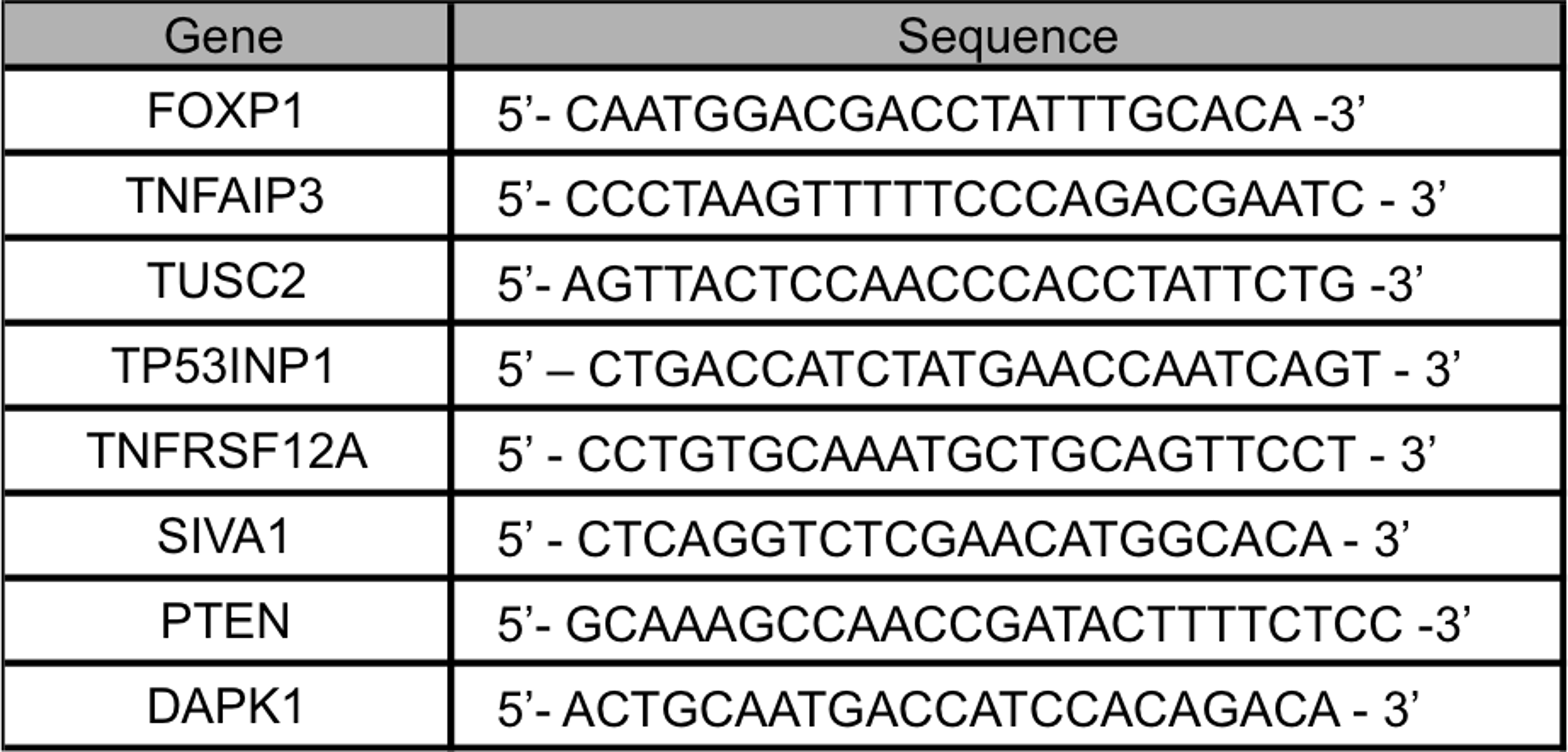

Supplement: S2 Table — (TIF) [file pone.0137887.s006.tif]
